# Supplementary material for: Impact of the announcement and implementation of the UK Soft Drinks Industry Levy on sugar content, price, product size and number of available soft drinks in the UK, 2015-19: A controlled interrupted time series analysis
Source: PLoS Med. 2020 Feb 11;17(2):e1003025. doi: 10.1371/journal.pmed.1003025 (PMC7012398; doi:10.1371/journal.pmed.1003025)
Supplement: S1 Appendix — (DOCX) [file pmed.1003025.s001.docx]

**The impact of the announcement and implementation of the UK Soft Drinks Industry Levy on sugar content, price, product size and number of available soft drinks in the UK, 2015-19: a controlled interrupted time series analysis**

# S1 Appendix: Analysis of impact of soft drinks industry levy on proportion of drinks over higher levy threshold (8g sugar per 100ml)

*Introduction:* The main paper reports results of models that measure the impact of the announcement and the implementation of the SDIL on the proportion of intervention and control drinks that are over the levy sugar threshold (5g sugar per 100ml). This appendix reports equivalent results for models that measure the impact of the SDIL on the proportion of drinks over the high levy threshold (8g sugar per 100ml).

*Methods:* The datasets used, definitions of key terms and descriptions of regression models used are reported in the main paper. The only difference is the threshold used for the binary variable in the logistic regression models – here the outcome variable is 1 if a drink contains greater than 8g sugar per 100ml and 0 otherwise. Additionally, we show trends in the raw data for both intervention and control drinks over both levy thresholds using stacked line charts.

*Results:* Table A compares the percentage of drinks over the high levy threshold with a counterfactual where the SDIL was not announced or implemented and Fig A shows the results for all intervention and control drinks. The percentage of intervention drinks over the high levy threshold reduced after the announcement of the SDIL only slowly at first, but with rapid changes just prior to the implementation. Just 50 days before the implementation, intervention drinks with enough sugar to be levied at the high rate had fallen by 20.0 (95% CI: 19.5, 20.5) percentage points – 50 days after implementation drinks levied at this rate had fallen by 28.7 (28.3, 29.0) percentage points. As of February 2019, there was a 31.3 (30.9, 31.6) percentage point fall, leaving only 7.1% (6.7%, 7.5%) of intervention drinks above the high levy threshold. Equivalent models for the control drinks found no impact of the announcement or implementation of the SDIL on percentage of drinks above the high levy threshold (p > 0.05 for all regression parameters). Due to a (non-significant) upwards trend in the percentage of control drinks above the high levy threshold before the announcement, the comparison with the counterfactual shows considerable falls (see Fig A). The pattern of sugar reduction in own-brand and branded drinks was very different – for own-brand drinks, sugar levels were already falling before the announcement of the SDIL, but these falls accelerated after the announcement. However, by the time of the implementation of the SDIL, only 3.5% (3.1%, 4.0%) of own-brand eligible drinks remained over the high levy threshold and further sugar reduction stalled. For branded drinks, there was a large fall in the proportion of drinks either side of the implementation of the levy, which had resulted in a 40.4 (40.0, 40.9) percentage point fall in the number of branded eligible drinks over the high levy threshold by February 2019.

**Table A: Difference between observed and counterfactual (extrapolation of pre-announcement trends) percentage of drinks over the high levy sugar threshold (>8g sugar per 100ml)**

|  | Difference in percentage^1^ of drinks over levy sugar threshold (95% confidence intervals) | | | |
| --- | --- | --- | --- | --- |
|  | 5^th^ May 2016  (50 days post-announcement) | 15^th^ February 2018 (50 days pre-implementation) | 26^th^ May 2018  (50 days post-implementation) | 17^th^ February 2019 (End of dataset) |
| **All intervention drinks** | -0.0 (-1.6, 0.7) | -20.0 (-20.5, -19.5) | -28.7 (-29.0, -28.3) | -31.3 (-31.6, -30.9) |
| Branded intervention drinks | -1.3 (-2.7, -0.0) | -24.7 (-25.3, -24.1) | -36.1 (-36.5, -35.7) | -40.4 (-40.9, -40.0) |
| Own-brand intervention drinks | 1.8 (-0.1, 3.8) | -9.6 (-10.1, -8.9) | -9.8 (-10.3, -9.3) | -7.7 (-8.2, -7.0) |
| **All control drinks** | 1.3 (-0.4, -3.0) | -11.9 (-12.7, -11.1) | -13.5 (-14.2, -12.7) | -15.6 (-16.5, -14.6) |

*^1^ Results are presented as percentage point differences compared to the counterfactual (extrapolation of pre-announcement trend).*

**Fig A: Change in proportion of drinks over the high levy threshold (8g sugar per 100ml), September 2015 to February 2019, intervention and control drinks, compared to counterfactual scenario of no SDIL**

**
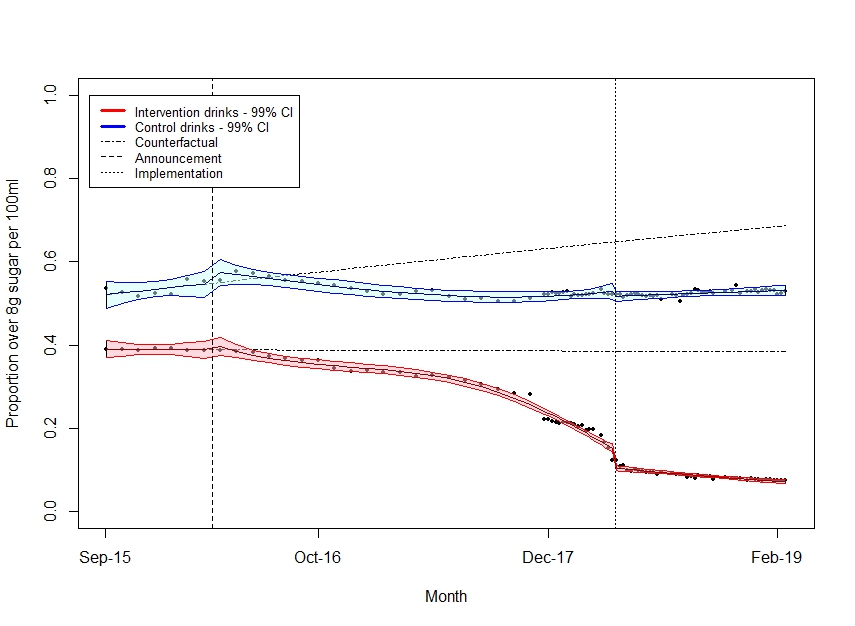
**

Fig B shows the proportion of both intervention and control drinks over each levy threshold and shows how the pace of reformulation of intervention drinks increased between the announcement and implementation of the SDIL.

**Fig B Trends in the proportion of intervention (eligible) and control (exempt) drinks over each levy threshold**

**
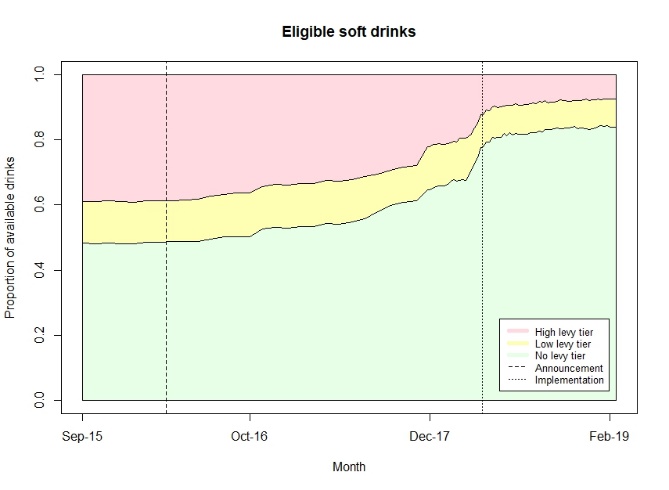

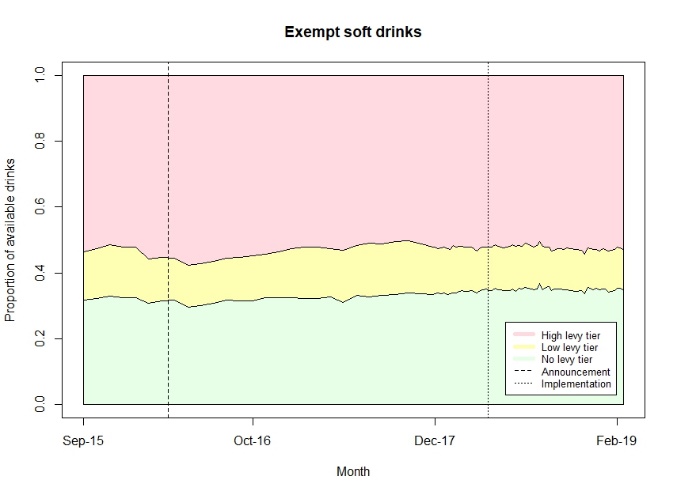
**
