# Supplementary material for: Impact of the announcement and implementation of the UK Soft Drinks Industry Levy on sugar content, price, product size and number of available soft drinks in the UK, 2015-19: A controlled interrupted time series analysis
Source: PLoS Med. 2020 Feb 11;17(2):e1003025. doi: 10.1371/journal.pmed.1003025 (PMC7012398; doi:10.1371/journal.pmed.1003025)
Supplement: S2 Appendix — (DOCX) [file pmed.1003025.s002.docx]

**The impact of the announcement and implementation of the UK Soft Drinks Industry Levy on sugar content, price, product size and number of available soft drinks in the UK, 2015-19: a controlled interrupted time series analysis**

# S2 Appendix: Model parameters for all models presented in the main analysis and supplementary material

*Parameter description:*

T Time, measured in 100 day units, with T = 0 at the point of the implementation of the SDIL (6^th^ April 2018)

A Dummy variable indicating the time between the announcement (16^th^ March 2016) and implementation (6^th^ April 2018) of the UK soft drinks industry levy (SDIL) – **This parameter estimates the level change due to announcement of the SDIL**

AT Interaction between A and T

ATT Interaction between A and T^2^

ATTT Interaction between A and T^3^ – **These parameters estimate the slope change due to the announcement of the SDIL**

I Dummy variable indicating the time after the implementation of the SDIL – **This parameter estimates the level change due to the implementation of the SDIL**

IT Interaction between I and T – **This parameter estimates the slope change due to the implementation of the SDIL**

Dec A dummy variable indicating the month of December

**Table B: Model parameters for all models reported in main paper**

| *Parameter* | *Effect size* | *95% confidence intervals* | *p* |
| --- | --- | --- | --- |
| **Above levy sugar threshold, intervention drinks** | | | |
| (Intercept) | -0.005 | (-0.504, 0.495) | 0.985 |
| T | -0.009 | (-0.067, 0.049) | 0.77 |
| A | -1.036 | (-1.538, -0.533) | <0.001 |
| AT | -0.462 | (-0.554, -0.370) | <0.001 |
| ATT | -0.083 | (-0.106, -0.059) | <0.001 |
| ATTT | -0.005 | (-0.008, -0.003) | <0.001 |
| I | -1.394 | (-1.895, -0.893) | <0.001 |
| IT | -0.089 | (-0.150, -0.027) | 0.005 |
| **Above levy sugar thresholds, control drinks** | | | |
| (Intercept) | 0.932 | (0.117, 1.747) | 0.025 |
| T | 0.020 | (-0.074, 0.115) | 0.673 |
| A | -0.304 | (-1.121, 0.513) | 0.465 |
| AT | -0.070 | (-0.206, 0.065) | 0.309 |
| ATT | -0.009 | (-0.041, 0.024) | 0.612 |
| ATTT | -0.001 | (-0.004, 0.002) | 0.629 |
| I | -0.312 | (-1.127, 0.504) | 0.454 |
| IT | -0.020 | (-0.117, 0.078) | 0.694 |
| **Price, all drinks, high levy category, intervention** | | | |
| (Intercept) | 3.243 | (3.224, 3.262) | <0.001 |
| T | 0.021 | (0.010, 0.031) | <0.001 |
| I | 0.029 | (-0.002, 0.059) | 0.064 |
| Dec | -0.024 | (-0.049, 0.002) | 0.068 |
| **Price, all drinks, low levy category, intervention** | | | |
| (Intercept) | 3.493 | (3.474, 3.512) | <0.001 |
| T | 0.019 | (0.009, 0.028) | <0.001 |
| I | -0.033 | (-0.063, -0.003) | 0.031 |
| Dec | -0.064 | (-0.089, -0.039) | <0.001 |
| **Price, all drinks, no levy category, intervention** | | | |
| (Intercept) | 2.630 | (2.621, 2.639) | <0.001 |
| T | 0.022 | (0.017, 0.027) | <0.001 |
| I | 0.025 | (0.011, 0.040) | <0.001 |
| Dec | -0.036 | (-0.049, -0.024) | <0.001 |
| **Price, all drinks, control** | | | |
| (Intercept) | 3.128 | (3.120, 3.137) | <0.001 |
| T | 0.006 | (0.002, 0.011) | 0.006 |
| I | -0.006 | (-0.020, 0.007) | 0.357 |
| Dec | 0.002 | (-0.009, 0.014) | 0.693 |
| **Price, branded drinks, high levy category, intervention** | | | |
| (Intercept) | 3.241 | (3.221, 3.260) | <0.001 |
| T | 0.021 | (0.010, 0.031) | <0.001 |
| I | 0.045 | (0.013, 0.076) | 0.005 |
| Dec | -0.023 | (-0.049, 0.004) | 0.09 |
| **Price, branded drinks, low levy category, intervention** | | | |
| (Intercept) | 3.549 | (3.531, 3.566) | <0.001 |
| T | 0.022 | (0.012, 0.031) | <0.001 |
| I | -0.051 | (-0.079, -0.023) | <0.001 |
| Dec | -0.068 | (-0.091, -0.045) | <0.001 |
| **Price, branded drinks, no levy category, intervention** | | | |
| (Intercept) | 2.810 | (2.801, 2.819) | <0.001 |
| T | 0.014 | (0.009, 0.019) | <0.001 |
| I | 0.015 | (0.001, 0.030) | 0.040 |
| Dec | -0.038 | (-0.05, -0.026) | <0.001 |
| **Price, branded drinks, control** | | | |
| (Intercept) | 3.297 | (3.288, 3.306) | <0.001 |
| T | 0.007 | (0.002, 0.012) | 0.004 |
| I | -0.015 | (-0.029, -0.001) | 0.036 |
| Dec | 0.004 | (-0.008, 0.015) | 0.539 |
| **Price, own-brand drinks, high levy category, intervention** | | | |
| (Intercept) | 3.311 | (3.228, 3.393) | <0.001 |
| T | 0.016 | (-0.017, 0.049) | 0.336 |
| I | -0.256 | (-0.368, -0.144) | <0.001 |
| Dec | -0.032 | (-0.124, 0.059) | 0.490 |
| **Price, own-brand drinks, low levy category, intervention** | | | |
| (Intercept) | 2.496 | (2.403, 2.590) | <0.001 |
| T | -0.017 | (-0.061, 0.026) | 0.433 |
| I | 0.451 | (0.312, 0.590) | <0.001 |
| Dec | 0.013 | (-0.102, 0.129) | 0.822 |
| **Price, own-brand drinks, no levy category, intervention** | | | |
| (Intercept) | 1.976 | (1.958, 1.994) | <0.001 |
| T | 0.028 | (0.018, 0.038) | <0.001 |
| I | -0.011 | (-0.040, 0.019) | 0.467 |
| Dec | -0.001 | (-0.025, 0.022) | 0.909 |
| **Price, own-brand drinks, control** | | | |
| (Intercept) | 2.508 | (2.495, 2.520) | <0.001 |
| T | 0.002 | (-0.005, 0.009) | 0.556 |
| I | 0.001 | (-0.019, 0.021) | 0.920 |
| Dec | 0.005 | (-0.012, 0.022) | 0.570 |
| **Product size, all drinks, high levy category, intervention** | | | |
| (Intercept) | 6.629 | (6.603, 6.656) | <0.001 |
| T | -0.048 | (-0.063, -0.033) | <0.001 |
| I | 0.001 | (-0.043, 0.046) | 0.960 |
| **Product size, all drinks, low levy category, intervention** | | | |
| (Intercept) | 6.455 | (6.436, 6.474) | <0.001 |
| T | 0.010 | (0.000, 0.021) | 0.059 |
| I | 0.020 | (-0.011, 0.051) | 0.210 |
| **Product size, all drinks, no levy category, intervention** | | | |
| (Intercept) | 6.922 | (6.914, 6.931) | <0.001 |
| T | -0.011 | (-0.016, -0.006) | <0.001 |
| I | -0.002 | (-0.017, 0.013) | 0.783 |
| **Product size, all drinks, control** | | | |
| (Intercept) | 6.580 | (6.572, 6.587) | <0.001 |
| T | -0.003 | (-0.007, 0.001) | 0.132 |
| I | 0.006 | (-0.006, 0.018) | 0.321 |
| **Product size, branded drinks, high levy category, intervention** | | | |
| (Intercept) | 6.635 | (6.608, 6.663) | <0.001 |
| T | -0.048 | (-0.063, -0.032) | <0.001 |
| I | -0.009 | (-0.055, 0.038) | 0.711 |
| **Product size, branded drinks, low levy category, intervention** | | | |
| (Intercept) | 6.432 | (6.413, 6.451) | <0.001 |
| T | 0.008 | (-0.003, 0.019) | 0.151 |
| I | 0.026 | (-0.006, 0.058) | 0.116 |
| **Product size, branded drinks, no levy category, intervention** | | | |
| (Intercept) | 6.890 | (6.880, 6.900) | <0.001 |
| T | -0.008 | (-0.014, -0.002) | 0.009 |
| I | 0.000 | (-0.018, 0.018) | 0.997 |
| **Product size, branded drinks, control** | | | |
| (Intercept) | 6.513 | (6.505, 6.522) | <0.001 |
| T | -0.003 | (-0.008, 0.002) | 0.281 |
| I | 0.009 | (-0.005, 0.023) | 0.207 |
| **Product size, own-brand drinks, high levy category, intervention** | | | |
| (Intercept) | 6.443 | (6.357, 6.529) | <0.001 |
| T | -0.058 | (-0.093, -0.023) | 0.001 |
| I | 0.249 | (0.129, 0.368) | <0.001 |
| **Product size, own-brand drinks, low levy category, intervention** | | | |
| (Intercept) | 6.864 | (6.815, 6.912) | <0.001 |
| T | 0.034 | (0.010, 0.058) | 0.006 |
| I | -0.157 | (-0.232, -0.082) | <0.001 |
| **Product size, own-brand drinks, no levy category, intervention** | | | |
| (Intercept) | 7.038 | (7.025, 7.051) | <0.001 |
| T | -0.020 | (-0.028, -0.012) | <0.001 |
| I | 0.005 | (-0.018, 0.028) | 0.661 |
| **Product size, own-brand drinks, control** | | | |
| (Intercept) | 6.836 | (6.827, 6.846) | <0.001 |
| T | -0.006 | (-0.012, -0.001) | 0.029 |
| I | 0.008 | (-0.008, 0.024) | 0.351 |
| **Number of products, all drinks, high levy category, intervention** | | | |
| (Intercept) | 279.9 | (261.5, 298.2) | <0.001 |
| T | -49.9 | (-73.4, -26.4) | <0.001 |
| I | -26.6 | (-48.3, -5.0) | 0.019 |
| IT | 46.6 | (22.3, 70.9) | <0.001 |
| **Number of products, all drinks, low levy category, intervention** | | | |
| (Intercept) | 299.5 | (289.0, 310.0) | <0.001 |
| T | -7.5 | (-13.9, -1.1) | 0.025 |
| I | -1.2 | (-19.7, 17.2) | 0.895 |
| **Number of products, all drinks, no levy category, intervention** | | | |
| (Intercept) | 2234.7 | (2161.0, 2308.4) | <0.001 |
| T | 30.9 | (-14.1, 75.9) | 0.184 |
| I | -54.3 | (-183.7, 75.2) | 0.415 |
| **Number of products, all drinks, control** | | | |
| (Intercept) | 2023.4 | (1920.4, 2126.4) | <0.001 |
| T | 93.5 | (-38.5, 225.5) | 0.170 |
| I | -49.5 | (-171.1, 72.1) | 0.428 |
| IT | -123.0 | (-259.6, 13.5) | 0.083 |
| **Number of products, branded drinks, high levy category, intervention** | | | |
| (Intercept) | 264.9 | (248.1, 281.7) | <0.001 |
| T | -34.9 | (-56.4, -13.4) | 0.002 |
| I | -25.5 | (-45.3, -5.6) | 0.014 |
| IT | 31.9 | (9.7, 54.2) | 0.007 |
| **Number of products, branded drinks, low levy category, intervention** | | | |
| (Intercept) | 272.0 | (262.6, 281.4) | <0.001 |
| T | -7.1 | (-12.9, -1.4) | 0.018 |
| I | 1.7 | (-14.8, 18.2) | 0.843 |
| **Number of products, branded drinks, no levy category, intervention** | | | |
| (Intercept) | 1811.6 | (1754.3, 1868.8) | <0.001 |
| T | 32.3 | (-2.6, 67.2) | 0.075 |
| I | -12.6 | (-113.2, 87.9) | 0.806 |
| **Number of products, branded drinks, control** | | | |
| (Intercept) | 1606.6 | (1524.1, 1689.0) | <0.001 |
| T | 86.3 | (-19.3, 191.9) | 0.114 |
| I | -34.7 | (-132, 62.6) | 0.487 |
| IT | -112.4 | (-221.7, -3.1) | 0.048 |
| **Number of products, own-brand drinks, high levy category, intervention** | | | |
| (Intercept) | 15.0 | (12.9, 17.1) | <0.001 |
| T | -15.0 | (-17.7, -12.3) | <0.001 |
| I | -1.2 | (-3.7, 1.3) | 0.352 |
| IT | 14.7 | (11.9, 17.5) | <0.001 |
| **Number of products, own-brand drinks, low levy category, intervention** | | | |
| (Intercept) | 24.2 | (21.2, 27.2) | <0.001 |
| T | -5.2 | (-9.1, -1.4) | 0.01 |
| I | -0.2 | (-3.7, 3.4) | 0.927 |
| IT | 5.2 | (1.2, 9.2) | 0.014 |
| **Number of products, own-brand drinks, no levy category, intervention** | | | |
| (Intercept) | 423.1 | (402.9, 443.2) | <0.001 |
| T | -1.4 | (-13.7, 10.9) | 0.821 |
| I | -41.6 | (-77.0, -6.3) | 0.024 |
| **Number of products, own-brand drinks, control** | | | |
| (Intercept) | 416.8 | (392.6, 441.0) | <0.001 |
| T | 7.2 | (-23.8, 38.2) | 0.652 |
| I | -14.8 | (-43.3, 13.8) | 0.315 |
| IT | -10.7 | (-42.8, 21.4) | 0.517 |
