# Supplementary material for: Impact of the announcement and implementation of the UK Soft Drinks Industry Levy on sugar content, price, product size and number of available soft drinks in the UK, 2015-19: A controlled interrupted time series analysis
Source: PLoS Med. 2020 Feb 11;17(2):e1003025. doi: 10.1371/journal.pmed.1003025 (PMC7012398; doi:10.1371/journal.pmed.1003025)
Supplement: S3 Appendix — (DOCX) [file pmed.1003025.s003.docx]

**The impact of the announcement and implementation of the UK Soft Drinks Industry Levy on sugar content, price, product size and number of available soft drinks in the UK, 2015-19: a controlled interrupted time series analysis**

# S3 Appendix: Comparison of foodDB and BrandView datasets

*Introduction:* This appendix provides a comparison of the two datasets that have been combined for analysis of the impact of the announcement and implementation of the Soft Drinks Industry Levy (SDIL) on the proportion of drinks over each levy threshold (see main paper and appendix 3) and on the mean sugar levels in drinks (see appendix 2).

*Methods:* We used data collected from the websites of the three leading UK supermarkets (Asda, Sainsbury’s and Tesco) that together account for 58% of UK grocery sales^[[1]](#footnote-1)^. One source was a web-scraping and data processing software platform called foodDB which was developed in-house by researchers in the Centre on Population Approaches for Non-Communicable Disease Prevention at the University of Oxford. Full details of the methods of data collection using foodDB are provided elsewhere^[[2]](#footnote-2)^. Briefly, foodDB collects data on over 99% of all food and drink products available for purchase on the supermarket websites each week, including product name, nutritional information, ingredients, product size, price and whether or not the product is on promotion.

To assess validity of the data collected by foodDB, a validation exercise was conducted that compared foodDB data with equivalent data collected from 295 randomly selected products in real life stores. Agreement between foodDB and the store sample for both sugar levels (g per 100g / 100ml) and price (£ per 100g / 100ml) was measured by assessing percentage agreement between the two sources and observing Bland-Altman plots for detection of bias. For percentage agreement, we rounded sugar levels to the nearest gram per 100g / 100ml, as this data was not always recorded to the same number of decimal places in each dataset.

For the controlled interrupted time series analyses, we used weekly data from foodDB from 26th November 2017 (its initial data collection period) until 17th February 2019, consisting of 58 time points (NB: 6 weeks of data collection were excluded from the dataset, due to errors with the data collection which resulted in collection of data on less than 90% of all observed drinks).

The second data source was the commercial company BrandView, which extracts data from products available in Asda, Sainsbury’s and Tesco. We purchased data corresponding to the first of every month between August 2015 and September 2018. The initial data point was dropped as data was available for less than 90% of all observed drinks, leaving 37 time points.

Both the foodDB and BrandView datasets were restricted to our definition of soft drinks (see main paper), and then categorised as intervention or control based on supermarket categorisation and manual inspection of product names, using similar code and methods for each dataset. We categorised drinks as ‘own-brand’ if the product name contained Asda, Tesco or Sainsbury’s, and as ‘branded’ otherwise.

To compare data collected from these two sources we compared drink categories, supermarkets, branded status, intervention / control status, price, product size and sugar level with Chi squared tests for categorical variables and Wilcoxon rank-sum tests for continuous variables. We observed trends in own-brand and branded products separately across the entire data collection period (including November 2017 – September 2018 where data were available from both sources). We plotted trends in the following variables: Number of products per data snapshot; mean sugar levels (g per 100ml); geometric mean price (p per 100ml); and geometric mean product size (ml). NB: geometric means were used for comparability with the main analyses, where these variables were log-transformed for the regression models.

*Results:* Of the 295 products identified in real-life stores and compared with equivalent products collected using foodDB, 193 had data for sugar levels in both datasets and 254 had data for price in both datasets. For sugar, 90.0% (95% confidence intervals 85.3%-93.9%) had the same sugar levels. For price, 77.6% (72.4%-82.7%) of the products matched between the online and real-life stores.

The Bland-Altman plots are shown in Figs C and D and demonstrate no evidence of systematic difference between the two data sources.

**Fig C Bland-Altman plot comparing price (p per 100g / 100ml) of 254 products identified in real-life stores and online**


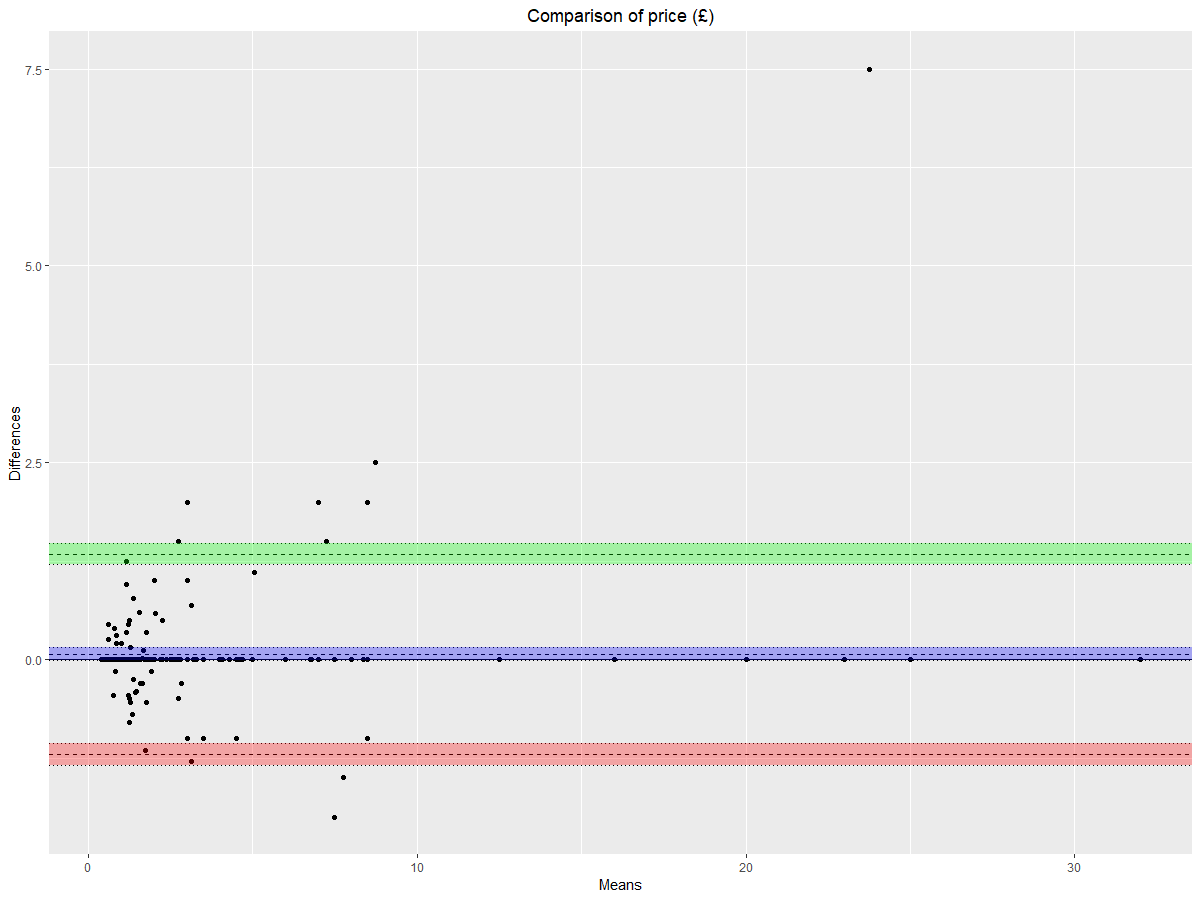


**Fig D Bland-Altman plot comparing sugar (g per 100g / 100ml) of 193 products identified in real-life stores and online**


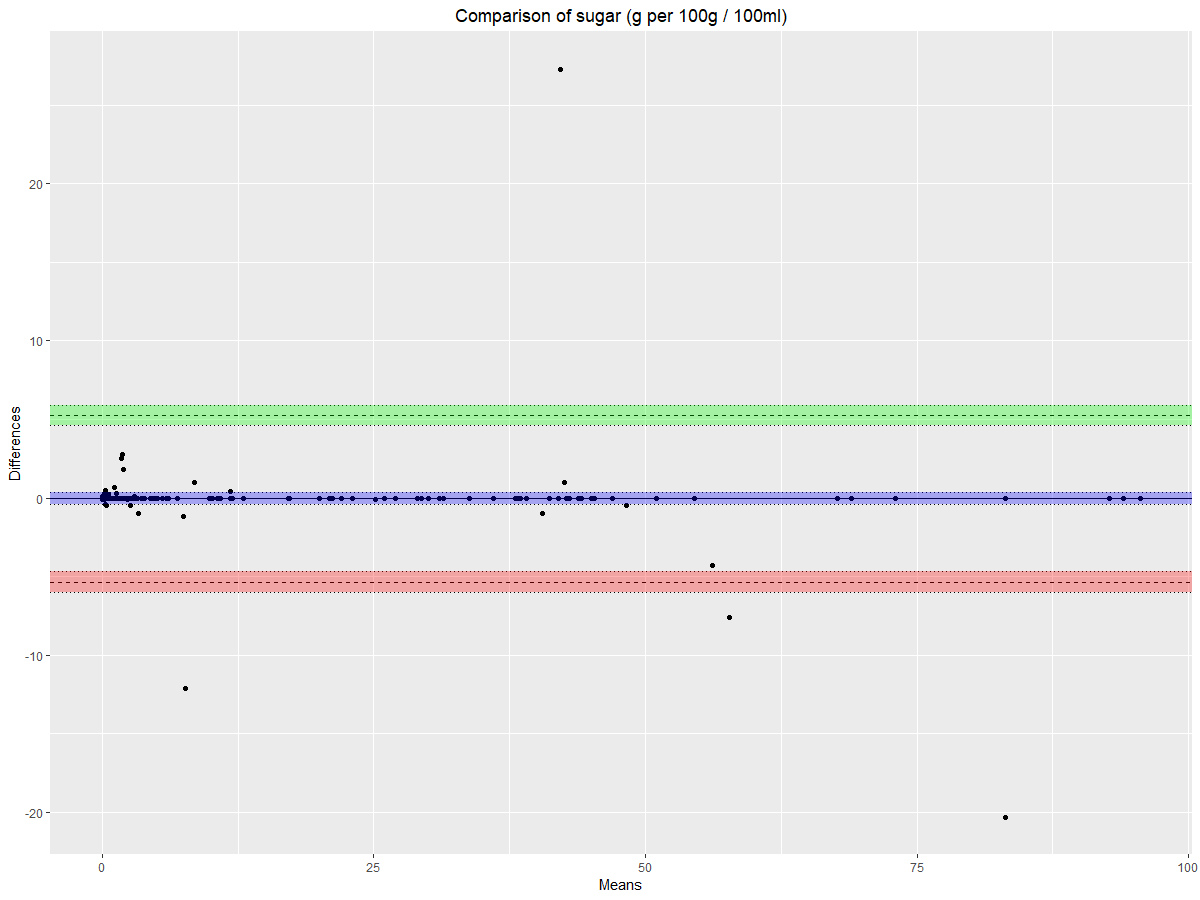


Table C shows descriptive statistics for the two datasets. There were differences between the two datasets in both the types of drinks observed and the amount of observations from each supermarket, with the foodDB dataset collecting data on similar number of drinks from each of the three supermarkets whereas BrandView predominantly provided data from Tesco. Splits between branded and own-brand status, and between intervention and control drinks were similar across the two datasets, but a greater proportion of branded products and control drinks were found in foodDB. There were small differences in the median price and sugar levels of drinks collected in the two datasets, which may reflect the different time periods over which data were collected.

**Table C Descriptive statistics comparing the foodDB and BrandView datasets**

| *Variable* | | *foodDB* | *BrandView* | *p^1^* |
| --- | --- | --- | --- | --- |
| Type of drink, n (%) | |  |  |  |
|  | Carbonate | 44,230 (30.7) | 33,323 (37.6) |  |
|  | Energy drink | 13,832 (9.6) | 3,434 (3.9) |  |
|  | Squash or cordial | 12,061 (8.4) | 7,887 (8.9) |  |
|  | Flavoured water | 8,539 (5.9) | 4,125 (4.7) |  |
|  | Milk-based drink | 20,157 (14.0) | 9,480 (10.7) |  |
|  | Fruit juice and smoothies | 45,253 (31.4) | 30,373 (34.3) | <0.001 |
| Supermarket, n (%) | |  |  |  |
|  | Tesco | 47,632 (33.0) | 51,254 (57.8) |  |
|  | Sainsbury’s | 50,069 (34.8) | 15,963 (18.0) |  |
|  | Asda | 46,371 (32.2) | 21,405 (24.2) | <0.001 |
| Branded status, n (%) | |  |  |  |
|  | Branded | 110,924 (77.0) | 63,460 (71.6) |  |
|  | Own-brand | 33,148 (23.0) | 25,162 (28.4) | <0.001 |
| SDIL status, n (%) | |  |  |  |
|  | Intervention | 89,175 (61.9) | 59,703 (67.4) |  |
|  | Control | 54,897 (38.1) | 28,919 (32.6) | <0.001 |
| Price £ per 100ml, median (IQR) | | 0.17 (0.10-0.27) | 0.15 (0.10-0.25) | <0.001 |
| Product size ml, median (IQR) | | 1000 (604-1500) | 1000 (500-1500) | 0.504 |
| Sugar g per 100ml, median (IQR) | | 4.6 (0.5-8.6) | 5.1 (0.5-10.0) | <0.001 |

*^1^ p values are derived from Chi-squared tests for categorical variables and Wilcoxon rank-sum tests for continuous variables*

Fig E shows overlapping trends in key variables between the two datasets. For mean sugar levels, price and product size there is strong agreement between the two datasets for own-brand products. However, for branded products there is less agreement. Mean product size and price were higher in branded foods (by 65ml and 0.99p per 100ml, respectively) in the foodDB dataset than in the BrandView dataset, and mean sugar levels were lower by 0.16g per 100ml.

Both the foodDB and BrandView datasets collected similar number of own-brand products at each data collection snapshot, but foodDB collected on average 225 more branded drinks per snapshot.

**Fig E Comparison of trend data in BrandView (Sep 15 – Sep 18) and foodDB (Nov 17 – Feb 19) for branded and own-brand products separately for A: number of drinks per snapshot, B: mean sugar level (g per 100ml), C: mean price (p per 100ml), D: mean product size (ml)**

**
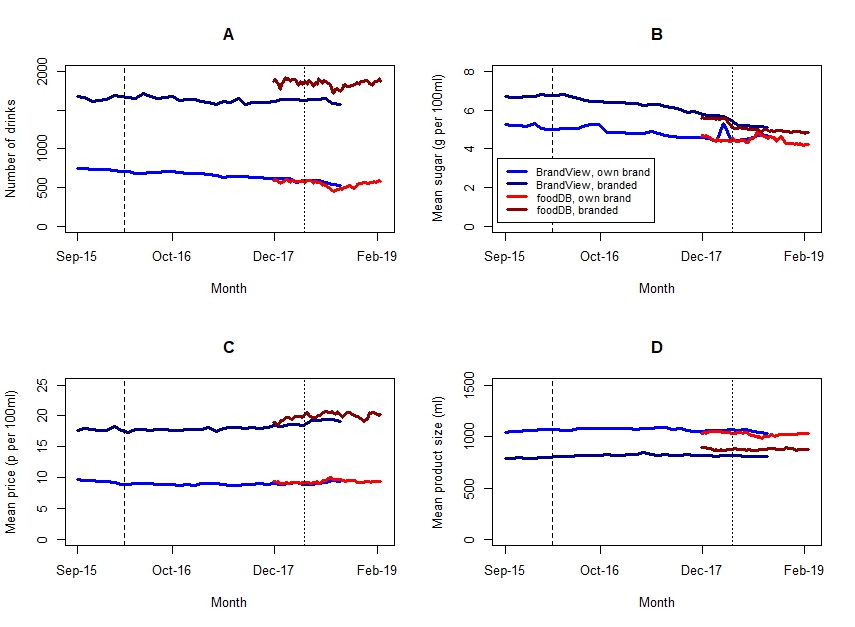
**

1. Kantar World Panel. Grocery market share UK. 12 weeks ending September 2018. Available at <https://www.kantarworldpanel.com/en/grocery-market-share/great-britain> Accessed 24th April 2019. [↑](#footnote-ref-1)
2. Harrington RA, Adhikari V, Rayner M, Scarborough P. Nutrient composition databases in the age of big data: foodDB, a comprehensive, real-time database infrastructure. *BMJ Open*, 2019 (in press). [↑](#footnote-ref-2)
