# Supplementary material for: Impact of the announcement and implementation of the UK Soft Drinks Industry Levy on sugar content, price, product size and number of available soft drinks in the UK, 2015-19: A controlled interrupted time series analysis
Source: PLoS Med. 2020 Feb 11;17(2):e1003025. doi: 10.1371/journal.pmed.1003025 (PMC7012398; doi:10.1371/journal.pmed.1003025)
Supplement: S4 Appendix — (DOCX) [file pmed.1003025.s004.docx]

**The impact of the announcement and implementation of the UK Soft Drinks Industry Levy on sugar content, price, product size and number of available soft drinks in the UK, 2015-19: a controlled interrupted time series analysis**

# S4 Pre-published protocol

The protocol for the full evaluation of the SDIL is available online here: <https://njl-admin.nihr.ac.uk/document/download/2010886>

The section relevant to the analyses reported in this paper is reproduced here.

**Study 1a: the impact of the SDIL on non-alcoholic drinks market diversity, total sugar content and price**

*Study design*

Using an in-house dataset collected from major UK supermarket websites, we will use interrupted time series (ITS) methods to study whether the implementation of the SDIL was associated with changes in level or trend of non-alcoholic drink market diversity, sugar content and price.

*Data source*

We will use an in-house, bespoke dataset (developed during our formative work) to assess non-alcoholic drink market diversification, formulation and price. We have developed automated data collection techniques (i.e. ‘data scraping’) and will use these to collect monthly, time-stamped data on all soft drinks available for purchase from six online UK supermarkets (Tesco, Morrison’s, Asda, Sainsbury’s, Waitrose and Ocado). Together these supermarkets (online and in-store) represent more than 75% of the UK grocery market.43 The resultant database (FoodDB) contains data on the complete product range of soft drinks from each supermarket in each month.

We will add data from any new online supermarkets that open during the project. Maintenance of FoodDB will be conducted monthly to ensure that the source code that supplies the dataset continues to run appropriately (it also will be necessary to adapt this source code when online supermarkets change their appearance, format or layout).

For each drink we will continue to collect: date of data collection; nutritional content; price; pack size; serving size; whether or not the drink is on promotion; and manufacturer. Complete datasets for all drinks were collected in December 2013 and October 2016. Data from 1281 Tesco drinks were collected from 2011 to 2016 using a combination of live and archived websites, 391 of which have more than three time points at which data was available. Full monthly data on all drinks from all six major online supermarkets is available from October 2016.

*Outcome measures*

We will have three outcome measures, one related to each of market diversity, formulation and price:

• number of products (e.g. Coca-Cola, not Coca-Cola 500ml bottle) available across six online supermarkets per month (market diversity)

• mean total sugar concentration in g/100ml per month (formulation)

• mean price (not sales-weighted) in £/100ml per month (price)

These will be considered overall and in each of the four drinks categories described above separately.

*Study period and sample size*

As described above we have some FoodDB data from 2013, with full data available from October 2016. This study will, therefore, include data from October 2016 (6 months after intervention announcement) – April 2020 (2 years after intervention implementation).

Data are available per calendar month, thus providing 12 time points/year from full establishment of FoodDB onwards (October 2016), and a total of 42 time points in the study. Currently, we estimate that more than 1000 unique soft drinks products will be included per time point. This dataset therefore substantially exceeds current recommendations for minimum samples sizes for ITS analyses of at least 10 time points before and after the intervention, and at least 100 observations per time point.

*Data analysis*

We will conduct single time point, ITS analyses for each outcome overall in each of the four drinks categories described above separately. The unit of analysis will be the calendar month. As data is only available from after intervention announcement to after intervention implementation, we will include only one ‘intervention’ point – intervention implementation.

As FoodDB is a database of products, rather than purchases or consumption, it will not be possible to study any differences by socio-demographic characteristics of purchases or consumers. Additional analyses stratified by supermarket price point may be possible e.g. as a student project add-on.
