# Supplementary material for: Impact of the announcement and implementation of the UK Soft Drinks Industry Levy on sugar content, price, product size and number of available soft drinks in the UK, 2015-19: A controlled interrupted time series analysis
Source: PLoS Med. 2020 Feb 11;17(2):e1003025. doi: 10.1371/journal.pmed.1003025 (PMC7012398; doi:10.1371/journal.pmed.1003025)
Supplement: S5 Appendix — (DOCX) [file pmed.1003025.s005.docx]

**The impact of the announcement and implementation of the UK Soft Drinks Industry Levy on sugar content, price, product size and number of available soft drinks in the UK, 2015-19: a controlled interrupted time series analysis**

# S5 Appendix: Analysis of impact of soft drinks industry levy on mean sugar levels

*Introduction:* This appendix reports results of a pre-determined analysis (see pre-published protocol <https://njl-admin.nihr.ac.uk/document/download/2010886>) of the impact of the announcement and implementation of the SDIL on mean sugar levels of intervention and control soft drinks in the UK.

*Methods:* We used the same dataset that was used for our analysis of the impact of the SDIL on the proportion of soft drinks above the levy sugar threshold, with the same definitions of intervention and control drinks. The main analysis is restricted to intervention drinks, with a second analysis using the same regression model but restricted to control drinks. Mean sugar levels over time were compared with a counterfactual which extrapolates trends from the period before the announcement of the SDIL. Full details of the datasets used, including definitions of key terms, can be found in the main manuscript.

The outcome variable for this analysis (sugar level of soft drinks) was not normally distributed in the dataset, nor did it follow a distribution that could be transformed to normality. Therefore, linear regression using the continuous variable was not appropriate. Instead, we collapsed the dataset into mean sugar levels (g per 100ml) for intervention and control drinks in the 85 time points between September 2015 and February 2019 available in the dataset. Linear regression models were then run against these collapsed data points.

Initial observation of the man sugar levels over time suggested non-linear trends between the announcement and implementation of the SDIL. Therefore, models were built allowing for polynomial trends during this period, with the final model selected on the basis of likelihood ratio tests comparing nested models using a threshold of p = 0.05 to decide whether adding extra polynomial order improved model fit sufficiently. This resulted in a cubic polynomial model fit between these points.

Although the continuous outcome variable used in the linear regression models (mean sugar levels) was not normally distributed, inspection of model residuals did not reveal any evidence of heteroscedasticity or deviation from normality.

We observed the distribution of sugar levels in drinks prior to the announcement of the SDIL and after its implementation to explore how manufacturers had reformulated their products.

*Results:* Fig F shows the trend in mean sugar levels of both intervention and control drinks over time. For intervention drinks, sugar levels were falling slowly before the announcement of the SDIL (p = 0.020), but accelerated after the announcement (p < 0.001), culminating in a substantial reduction in sugar levels just before the implementation of the SDIL (p < 0.001). After the implementation, the trend in sugar levels returned to a level that was not different to pre-announcement trends (p = 0.666). By February 2019, mean sugar levels in intervention soft drinks were lower than the counterfactual scenario of no SDIL by 2.13g per 100ml (95% CI: 2.08, 2.18). The control analysis found no evidence of difference in trends due to either the announcement or the implementation of the SDIL.

**Fig F Mean sugar levels (g per 100ml) for eligible and exempt drinks, September 2015 – February 2019**


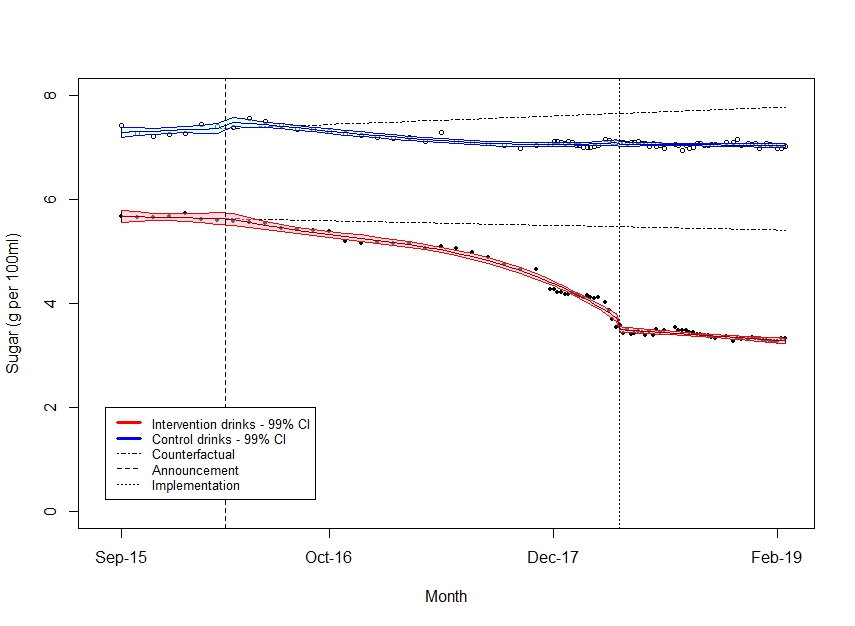


Fig G shows histograms of the sugar levels in observations of drinks before the announcement of the SDIL and after its introduction. Prior to the announcement of the SDIL, there is a large spike of drinks with less than 1g sugar per 100ml, but sugar levels in drinks are fairly even elsewhere. After the implementation of the SDIL, there is another large spike in drinks with sugar levels between 4.5g and 5.0g sugar per 100ml, suggesting that manufacturers reacted to the levy by removing just enough sugar from drinks to avoid the levy.

**Fig G Sugar levels (g per 100ml) in observations of drinks prior to the announcement of the SDIL and post-implementation of the SDIL**

**
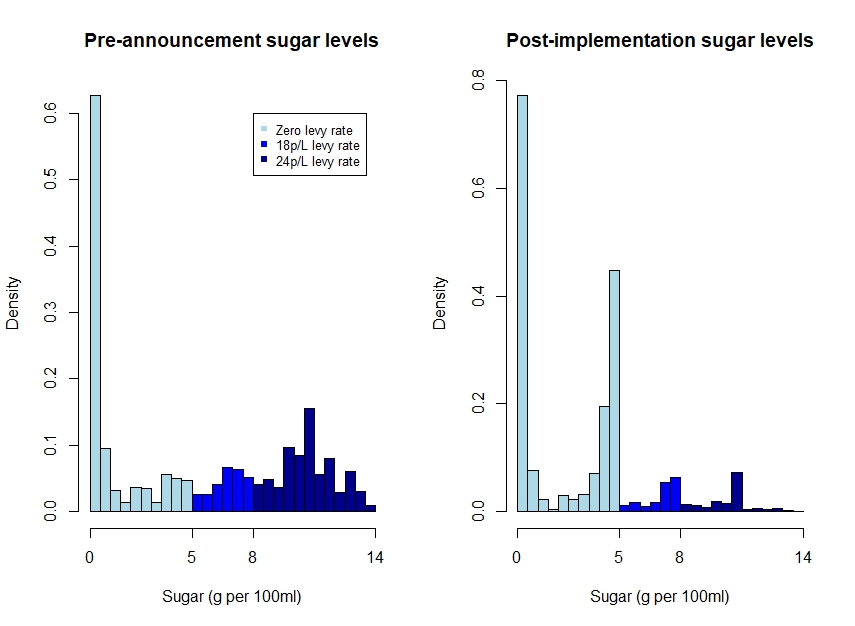
**
