# Supplementary material for: Impact of the announcement and implementation of the UK Soft Drinks Industry Levy on sugar content, price, product size and number of available soft drinks in the UK, 2015-19: A controlled interrupted time series analysis
Source: PLoS Med. 2020 Feb 11;17(2):e1003025. doi: 10.1371/journal.pmed.1003025 (PMC7012398; doi:10.1371/journal.pmed.1003025)
Supplement: Attachment — Submitted filename: scarborough.pdf [file pmed.1003025.s006.pdf]

This paper uses data from UK supermarkets to estimate the effect of the sugar levy.

I was asked for a statistical report and I interpret that to include all aspects of the design and conduct of the study.

1 Points of detail

Page 3 5− < 8 looks rather inelegant. 5 to 8? Between 5 and 8?

Page 7 I am afraid the last sentence on this page starting 'We included drinks' left me baffled. I am not sure what it means or what the implications are.

Page 8 Three supermarkets are mentioned here but on page 9 we find there were actually six. Which is correct?

Page 10 Again I am afraid I am baffled here. Why assign a drink to its last classification irrespective of changes in sugar content? I thought the whole point of the levy was to encourage movement (downwards). So would it not be better to have the classification time varying?

Page 12 I do not understand why the government chose to target drinks with less sugar (intervention) than the controls. This cannot be a typo as it is confirmed in Figure 1 on page 14. Perhaps this is worth a comment?

Page 13 We only see p–values here which is undesirable especially for the ones which fail to reach some level of statistical significance. Editors of health journals started calling for measures of effect size instead of p–values three decades ago (Langman, 1986; Gardner and Altman, 1986)

Page 14 My bafflement about page 10 is increased here. If you classify drinks by their status at the end then how do you interpret these results?

Page 19 Perhaps first to our knowledge would be better?

Page 20 Is there any data about exactly how much of the market is accounted for by on–line supermarket sales to reassure the reader about the possible bias discussed here? If there is, does that vary by socio–economic status?

Supplementary material S4 It would be better to give confidence intervals and for the ones which come from logistic regression to convert them to odds ratios. I know that with the information given the reader can do that but it is surprising how many people do not know how to.

2 Points of more substance

2.1 Data–driven modelling

Exclusion of data

In three places on pages 8 to 10 the authors exclude observations. There is no justification offered for this except that in some unspecified sense they were outlying. This seems undesirable without some account of why they are believed to be drawn from a contaminating population and without any picture of how much their exclusion affects the results.

How should time be in the model?

The authors have undertaken substantial data–driven selection of terms to go into their model. This is stated to have been guided by likelihood ratio tests although as far as I can see these are not presented anywhere. Even if we agree that time is unlikely to have affected outcomes in a linear fashion then what justification is there for choosing to use a cubic? If there is a scientific justification for supposing that the effect behaves in that way then does it make scientific sense to fit a cubic in time for part of the time period and linear elsewhere? Is there a scientific reason to suppose that the course over time is so different for the different outcomes that some should be linear rather than cubic?

December

Some of the models contain a dummy for December. Why was this thought a good idea? Why should manufacturers alter the formulation of their products for December and if they do why does this only affect some outcomes??

2.2 Overall comment on the models

There must be a concern here about the nature of this modelling. Selecting a subset of variables in a way driven by the data leads to a model which is unlikely to replicate (Babyak, 2004) and the same applies in an extended way to other data–driven choices. Although the authors have set out what they did it is unclear, at least to me, what the scientific justification was for most of the decisions.

3 Summary

Quite a lot is unclear to me and I find the modelling presentation has the flavour of a set of arbitrary choices rather than a coherent strategy.

Michael Dewey

References

M A Babyak. What you see may not be what you get: a brief, nontechnical introduction to overfitting in regression–type models. *Psychosomatic Medicine*, 66:411–421, 2004.

M J Gardner and D G Altman. Confidence intervals rather than P values: estimation rather than hypothesis testing. *British Medical Journal*, 292: 746–750, 1986.

M J S Langman. Towards estimation and confidence intervals. *British Medical Journal*, 292:716, 1986.
