# Supplementary material for: Impact of the announcement and implementation of the UK Soft Drinks Industry Levy on sugar content, price, product size and number of available soft drinks in the UK, 2015-19: A controlled interrupted time series analysis
Source: PLoS Med. 2020 Feb 11;17(2):e1003025. doi: 10.1371/journal.pmed.1003025 (PMC7012398; doi:10.1371/journal.pmed.1003025)
Supplement: Attachment — Submitted filename: Impact of SDIL on soft drinks_rebuttal_SEP2019.docx [file pmed.1003025.s007.docx]

Impact of the announcement and implementation of the UK Soft Drinks Industry Levy on sugar content, price, product size and number of available soft drinks in the UK, 2015-19: controlled interrupted time series analysis

Response to reviewers

Note for editors

We thank the editors for returning this manuscript to us so swiftly and for the helpful comments aimed at improving the manuscript. Our responses to all of the comments from the editors and the three reviewers are provided below, and an updated version of the manuscript, supplementary material and figures have been submitted.

Since submission of this manuscript, a new Government has been formed and the Soft Drinks Industry Levy (SDIL) has come under renewed scrutiny. The Government’s special health policy adviser, William Warr, has attacked the SDIL (Warr, 2019; Oliver, 2019) and the incoming Chief Medical Officer, Prof Chris Whitty, has been asked for a rapid review of the evidence surrounding sugar drink taxation. It is thought that this review is required before the end of the year. Public Health England have been asked to provide evidence to contribute to this review and have contacted us (as the NIHR-funded SDIL evaluation team) to ask for evidence of its effectiveness. This manuscript is an important contribution to this evaluation and we hope that it can be published in time to be included in the Whitty review. Whilst we understand that the peer review process must be given time to reach a conclusion that all reviewers and editors are satisfied with, we hope that the editors will consider fast-tracking decisions on this paper.

Oliver D. Health policy advice in the new number 10. *BMJ,* 2019;366:l5309. <https://www.bmj.com/content/366/bmj.l5309.full>

Warr W. Will our next PM be bold enough to tackle the biggest challenge facing the NHS? *Telegraph,* 19 July 2019. <https://www.telegraph.co.uk/politics/2019/07/19/will-next-pm-bold-enough-tackle-biggest-challenge-facing-nhs/>

Editor comments

If a prospective analysis plan (from your funding proposal, IRB or other ethics committee submission, study protocol, or other planning document written before analyzing the data) was used in designing the study, please include the relevant prospectively written document with your revised manuscript as a Supporting Information file to be published alongside your study, and cite it in the Methods section. A legend for this file should be included at the end of your manuscript. If no such document exists, please make sure that the Methods section transparently describes when analyses were planned, and when/why any data-driven changes to analyses took place. In either case, changes in the analysis—including those made in response to peer review comments—should be identified as such in the Methods section of the paper, with rationale.

*Response:* The protocol for the full Soft Drinks Industry Levy (SDIL) evaluation (for which this analysis constitutes one element) is available online and referenced in the document. The section of the protocol relevant to this analysis has now been reproduced in the supplementary material. There is a section in the methods titled ‘Changes to published protocol’ outlining any deviations.

Abstract- Please explain what is meant by “209,637 observations of soft drinks at 85 time points”

*Response:* We have amended this to make it clearer that there were a total of 209,637 observations in the full dataset, rather than at each time point. The sentence now reads: “We analysed data on a total of 209,637 observations of soft drinks over 85 time points between September 2015 and February 2019”.

Abstract-Please consider introducing the terms lower levy, higher levy and no levy categories earlier in the abstract.

*Response:* We have amended the abstract to include these definitions in the introduction section.

Abstract-Please quantify the main results (with 95% CIs and p values).

*Response:* We have added p values to the main results in the abstract

At this stage, we ask that you include a short, non-technical Author Summary of your research to make findings accessible to a wide audience that includes both scientists and non-scientists. The Author Summary should immediately follow the Abstract in your revised manuscript. This text is subject to editorial change and should be distinct from the scientific abstract. Please see our author guidelines for more information: https://journals.plos.org/plosmedicine/s/revising-your-manuscript#loc-author-summary

*Response:* This has been added to the manuscript.

Abstract-In the last sentence of the Methods and Findings section, please describe the main limitation(s) of the study's methodology

*Response:* We have added the sentence “These results are not sales weighted, so do not give an account of how sugar consumption from drinks may have changed over the time period.”

DAS- Note that a study author cannot be the contact person for the data. Please deposit your data with a third party such as a research ethics committee or data committee for data access by third parties and provide this information within the data availability statement

*Response:* Unfortunately we cannot release the full dataset due to legal restrictions. The data were acquired from supermarket websites without the express permission from the supermarkets themselves. If these data were to be used for commercial purposes then the University of Oxford would be in breach of UK copyright laws and liable for legal action from the supermarkets. Therefore, we are only able to release redacted versions of the dataset to a data archive, which we intend to do once the manuscript has been accepted for publication. We have clarified this situation with a revised DAS: “Data cannot be shared publicly because of legal restrictions to the use of copyrighted material for research purposes. Data use for this manuscript is covered by the Intellectual Property Office's Exceptions to Copyright for Non-Commercial Research and Private Study (https://www.gov.uk/guidance/exceptions-to-copyright). Data can only be shared under a licensed agreement that they will not be used for commercial purposes. Please contact the authors if you wish to access the data under licence. The Brand View dataset purchased and used for the analyses in this paper can be requested from the authors for the purpose of verification and replication of findings only. The dataset can be shared under licence which will restrict use of the dataset to this purpose only. An anonymised version of the foodDB dataset, where supermarket and product names are redacted, will be made available from the Oxford University Research Archive (https://ora.ox.ac.uk/) upon publication of this manuscript.”

Introduction-Line 82 please introduce the NHS on first view.

*Response:* This has been amended.

Page 9 Line 189-190 please clarify why these time points were excluded from analysis.

*Response:* This has been amended in a revised and restructured methods section, which includes a new data flowchart.

Line 221- please clarify what is meant by reasonably normally distributed and if linear regression models were appropriately used.

*Response:* This has been amended in the revised methods section, which now includes the following statement: “Visual inspections of p-p plots suggested that the price variable was not normally distributed and contained a long tail of high priced drinks. To convert to normality, we first excluded outlying drinks with a price greater than £1 per litre and then log-transformed the variable. We conducted linear regression modelling on the log-transformed price variable.”

Line 360 Please clarify what is meant by “obvious changes in the number of soft drinks available” and clarify in the methods section how this was evaluated.

*Response:* This has been amended in the revised manuscript to remove the word ‘obvious’.

Line 363- Please clarify if the speed of reformulation was directly tested in this study or consider toning down this sentence

*Response:* This sentence refers to the change in the slope of the line (which measures rate of change over time i.e. speed) after the announcement of the SDIL, compared to the pre-SDIL trend which is directly tested in the analysis.

Line 385-Please alter all assertions of primacy to “to the best of our knowledge”

*Response:* The manuscript has been amended accordingly.

Your study is observational and therefore causality cannot be inferred. Please remove language that implies causality, such as [ ]. Refer to associations instead

*Response:* The manuscript has been amended throughout accordingly.

Reviewer 1

This paper uses data from UK supermarkets to estimate the effect of the sugar levy. I was asked for a statistical report and I interpret that to include all aspects of the design and conduct of the study.

Points of detail

Page 3: 5-< 8 looks rather inelegant. 5 to 8? Between 5 and 8?

*Response:* This has been changed to ‘5 to 8’.

Page 7 I am afraid the last sentence on this page starting `We included drinks' left me baffled. I am not sure what it means or what the implications are.

*Response:* We have edited that sentence to make it clearer. The intention of this paragraph is to make it clear that soft drinks that are not subject to exemptions, but have sugar levels less than 5g per 100ml (e.g. Diet Coke, Pepsi Max) are included in the ‘intervention’ category rather than the ‘control’ category, and providing a justification for this decision. The re-edited section now reads as follows: “The decision regarding how to categorise soft drinks that are neither subject to exemptions, nor have sugar levels above the minimum threshold of 5g/100ml is not straightforward. These drinks are not subject to the levy, so could be regarded to be equivalent to drinks from exempt categories. However, we included such drinks in the intervention series as manufacturers could react to the SDIL by reducing sugar content of drinks, thereby moving drinks from categories that are taxed into categories that are not. If our study design included these non-taxed categories in the control series then we would allow drinks to migrate from the intervention to the control series over time, which would violate our assumption that the SDIL does not affect the control series.”

Page 8 Three supermarkets are mentioned here but on page 9 we find there were actually six. Which is correct?

*Response:* We have made considerable amendments to the methods section, as it was clear from the reviewers’ comments that the original manuscript was confusing. We apologise for this and hope the revisions are helpful. With regard to this specific point, we have added a new figure showing a data flowchart, which should clarify the sources of data for the separate analyses, and why some analyses used data from three supermarkets and some from six.

Page 10 Again I am afraid I am baffled here. Why assign a drink to its last classification irrespective of changes in sugar content? I thought the whole point of the levy was to encourage movement (downwards). So would it not be better to have the classification time varying?

*Response:* For the reformulation analysis, we always used the sugar content of the drink at the time of observing the drink. However, for both the price and product size analyses, this approach would be problematic as we display the results categorised by whether drinks are in the high levy, low levy or no levy category, so we categorise the drinks in the levy category from their final observation. We have restructured the ‘statistical methods’ section, so that each of the analyses has its own section, which we hope clarifies this situation.

Page 12 I do not understand why the government chose to target drinks with less sugar (intervention) than the controls. This cannot be a typo as it is confirmed in Figure 1 on page 14. Perhaps this is worth a comment?

*Response:* The comparison between median sugar levels in the control and intervention series, and the proportion of drinks over the minimum sugar threshold only provides part of the picture. In contrast to the intervention drinks, the control drinks (100% fruit juices and milk-based drinks) do not tend to contain any artificial sweeteners and as such they do not contain many zero sugar drinks. The distribution of sugar levels in control drinks and intervention drinks before the announcement of the SDIL is different (see figure below), with the intervention drinks having a spike at zero, and then a fairly uniform distribution compared with the control drinks, which have a fairly uniform distribution, with some grouping around 10g sugar per 100ml. Whilst we think these differences are interesting, we do not think there is room to give them ample discussion in an already crowded manuscript.

Page 13 We only see p values here which is undesirable especially for the ones which fail to reach some level of statistical significance. Editors of health journals started calling for measures of effect size instead of p values three decades ago (Langman, 1986; Gardner and Altman, 1986)

*Response:* On page 13, all of the reported results (both in the narrative and in table 2) use effect sizes and 95% confidence intervals rather than p values, with the exception of one statement “Equivalent models for the control drinks found no impact of the announcement or implementation of the SDIL on percentage of drinks above each levy threshold (p > 0.05 for all regression parameters – see S4 appendix)”, which we assume the reviewer is referring to. We agree that this statement cannot be supported solely by p values being over an arbitrary threshold, so have amended it to read as follows: “Equivalent models for the control drinks found little evidence of impact of the announcement or implementation of the SDIL on percentage of drinks above each levy threshold (for example, p > 0.05 for all regression parameters – see S4 appendix for all model results).”

Page 14 My bafflement about page 10 is increased here. If you classify drinks by their status at the end then how do you interpret these results?

*Response:* We assume that the reviewer is referring to figure 1, which would not make sense if all drinks were categorised by their sugar content at the end of the analysis. We hope this confusion has been addressed by our response to the earlier comment.

Page 19 Perhaps first to our knowledge would be better?

*Response:* Thank you – we have amended this statement accordingly.

Page 20 Is there any data about exactly how much of the market is accounted for by online supermarket sales to reassure the reader about the possible bias discussed here? If there is, does that vary by socioeconomic status?

*Response:* Unfortunately, we are not aware of any data sources that quantify the amount of sales of soft drinks that are from online supermarkets. There are estimates of the size of the online grocery market place, which accounts for 8% of all grocery sales in the UK (DEFRA, 2019). However, even if these data were available they would not be directly relevant to the analyses presented in this manuscript, which are based on soft drink *availability* rather than soft drink *sales.* Ideally, we would have an estimate of the amount of soft drinks that are available for purchase in the UK and are included in the datasets that we used for this analysis. We are not aware of any estimates of this quantity.

DEFRA. *Food statistics pocketbook.* DEFRA: London, 2019.

Supplementary material S4 It would be better to give confidence intervals and for the ones which come from logistic regression to convert them to odds ratios. I know that with the information given the reader can do that but it is surprising how many people do not know how to.

*Response:* For table S2 we have replaced the standard error with 95% confidence intervals throughout. However, we have not converted the effect size estimates with odds ratios for the results of logistic regression models. This is because the primary reason for providing the model parameters is to allow skilled readers to reproduce the models and hence estimate the difference between modelled results and counterfactual results at any time point that they choose. For this purpose, the effect sizes (i.e. model parameters) are more useful than the transformed odds ratios.

*Points of substance*

2.1 Data-driven modelling

Exclusion of data: In three places on pages 8 to 10 the authors exclude observations. There is no justification offered for this except that in some unspecified sense they were outlying. This seems undesirable without some account of why they are believed to be drawn from a contaminating population and without any picture of how much their exclusion affects the results.

*Response:* We hope the restructured methods and the addition of the data flowchart help to resolve this issue. Additionally, we have added the following text to the manuscript: “Visual inspections of p-p plots suggested that the price variable was not normally distributed and contained a long tail of high priced drinks. To convert to normality, we first excluded outlying drinks with a price greater than £1 per litre and then log-transformed the variable. We conducted linear regression modelling on the log-transformed price variable.”

How should time be in the model? The authors have undertaken substantial data-driven selection of terms to go into their model. This is stated to have been guided by likelihood ratio tests although as far as I can see these are not presented anywhere. Even if we agree that time is unlikely to have affected outcomes in a linear fashion then what justification is there for choosing to use a cubic? If there is a scientific justification for supposing that the effect behaves in that way then does it make scientific sense to fit a cubic in time for part of the time period and linear elsewhere? Is there a scientific reason to suppose that the course over time is so different for the different outcomes that some should be linear rather than cubic?

*Response:* We agree with the reviewer that we have not been transparent enough about the motivation for our modelling strategy, which was clearly data driven. We have added the following text to clarify: “We used a data-driven approach to build regression models with the aim of reproducing time trends observed in the datasets and isolating the impact of the announcement and implementation of the SDIL. For each analysis, we first observed trends in the non-modelled data which was used to inform the model building strategy. Where non-linear trends were observed, we included polynomial regression parameters, testing each additional parameter for improved model fit using likelihood ratio tests. Because of the very large number of possible models that could be tested, we restricted exploration of non-linear effects only to time periods where trends in the non-modelled data clearly deviated from linearity.” We have not presented results of the likelihood ratio tests as 61 were conducted throughout the analysis, and we believe presenting all of these results would be unnecessary. Interested readers can request these results from the corresponding author.

December: Some of the models contain a dummy for December. Why was this thought a good idea? Why should manufacturers alter the formulation of their products for December and if they do why does this only affect some outcomes??

*Response:* We hope that the newly restructured methods chapter will make this clearer. The dummy variable for December was only included for the analysis of the price of soft drinks, as supermarkets tend to lower the price of soft drinks in December for the Christmas period. As the reviewer suggests, there is no evidence that manufacturers would alter formulation, product size or number of drinks in the marketplace during December so this dummy variable was not included in those analyses.

2.2 Overall comment on the models

There must be a concern here about the nature of this modelling. Selecting a subset of variables in a way driven by the data leads to a model which is unlikely to replicate (Babyak, 2004) and the same applies in an extended way to other data-driven choices. Although the authors have set out what they did it is unclear, at least to me, what the scientific justification was for most of the decisions.

*Response:* We thank the reviewer for the reference to this paper. We agree that the data-driven approaches used here, which were designed to replicate trends observed in the UK, may limit the generalisability of our results to other jurisdictions that may introduce a similarly structured levy in the future. This is a limitation that we have now added to the discussion with the following statement: “The data-driven approaches that we have used for the modelling strategy may lead to over-fitted models which can limit the generalisability of these results to other jurisdictions considering introducing a similarly structured levy (Babyak, 2004).” However the central purpose of this analysis was not to predict the impact of future taxes, but to evaluate the impact of the UK SDIL, for which we believe these methods were appropriate.

3 Summary

Quite a lot is unclear to me and I find the modelling presentation has the flavour of a set of arbitrary choices rather than a coherent strategy.

*Response:* We hope that the restructured methods section and our responses to these comments have helped the clarity and coherence of the paper.

Reviewer 2

This very well-written paper describes changes in soft drink sugar content, product size, and availability in the UK after the announcement and implementation of the UK SDIL. The findings make an important contribution to the field of public health and are particularly important as the first evidence of the impact of tiered taxes on beverage industry behaviors.

*Response:* Thank you for your kind comments.

Introduction:

Page 4 - line 92: Many SSB taxes globally ,and all SSB taxes passed recently in the U.S. (Philadelphia, Berkeley, Oakland, San Francisco, Seattle, West Virginia, Boulder) are excise taxes levied on quantity - not ad valorem taxes. These recent taxes are expected to raise shelf prices, but also aim to generate revenue for local jurisdictions. The important distinction seems to be the SDIL's attempt to encourage product reformulation.

*Response:* The reviewer is absolutely correct – apologies for this error*.* The manuscript has been amended to refer to previous taxes as excise taxes.

Methods:

Include the number of unique products available in methods - I assume observations represents the total observations but it's not clear how many unique products this represents.

*Response:* Unfortunately it is not possible to estimate the total number of unique products in the dataset. This is partially due to theoretical challenges – what constitutes a unique soft drink? Are Coca-Cola 330ml, Coca-Cola 1L, Coca-Cola 6x330ml unique products? For the purpose of the reformulation analysis, probably not. For the purpose of the price and product size analyses they probably are. There is also a practical challenge. The datasets that we use for this analysis do not have a unique identifier field (e.g. barcodes), which means we can only link soft drinks by name which is an imprecise method. We have described this issue in the methods: “We had no unique identifier for the soft drinks that were included in the analysis, and therefore we were not able to link all observations at different time points. Therefore, we were unable to create a panel series, and structured our dataset as a repeat cross-sectional design.”

Lines 211-12: clarify that proportion over levy threshold limited to smaller (Brandview) dataset (I believe this is the case)

*Response:* In response to reviewer 1 we have amended the methods section to make descriptions of the separate analyses clearer. We have also added a data flowchart to clarify which data were used for each analysis.

Lines 212-217: clarify if "single item" is equivalent to non-multipack

*Response:* Throughout the analyses, we have included drinks sold individually and in multipacks separately. We have clarified this in the revised methods section.

Lines 222-224: clarify if clustering by unique product was accounted for in models and provide rationale for approach

*Response:* Clustering by unique products was not possible (see response to previous comments). We have made this clear in the revised methods section.

Results:

Table 1: explain "Number per week"- not clear what this represents (I assume unique products but N is then confusing)

*Response:* We have added an extra footnote to the table to clarify the meaning: “For ‘number per week’, all observations are collapsed in each time point, so this represents the number of time points in the analyses.”

Line 282: provide baseline % above threshold in text

*Response:* We would rather not provide this information in the text (it is available from the figures) as providing this baseline information will imply that the results are compared to a counterfactual of no change from baseline, whereas in fact the results are compared with a counterfactual of continuation of pre-SDIL trend.

Table 2: include baseline % above threshold

*Response:* For similar reasons, we do not think this information is helpful for interpreting the results of the paper.

Lines 311-314 and Table 3: It would be interesting to know how price changed among lower levy tier drinks that did not change category over time (if not different from results presented, would be useful to simply state that). It seems that results as presented would reflect reduction in price related to moving from higher to lower levy tier.

*Response:* The results as presented are *not* confounded by drinks moving from high levy to low levy categories over time (and hence being included in different regression models at different time points). We protected against this by matching drinks by name and categorising each drink by the levy category that it was in *after* implementation of the levy. In this way, we are only interested in changes of price of a defined group of soft drinks, rather than change in price of a set of soft drinks that changes over time. This has now been emphasised in the restructured methods section.

Appendix:

For S2, in text, provide "baseline" mean sugar content among eligible soft-drinks

*Response:* Again, for the same reasons as described above, we do not think it is helpful to provide this information due to the implication about what is used as the counterfactual.

Figure S4 vs. text: text states mean sugar content was 2.13 in Feb 2019 but in figure final mean sugar content appears to be above 3 g/100ml. Clarify discrepancy.

*Response:* In the text it states “By February 2019, mean sugar levels in eligible soft drinks were 2.13g per 100ml (95% CI: 2.08, 2.18) lower than in the counterfactual scenario of no SDIL” – as the reviewer suggests the mean sugar level at the end of the dataset was closer to 3g per 100ml. We have reworded this so the meaning is absolutely clear: “By February 2019, mean sugar levels in intervention soft drinks were lower than the counterfactual scenario of no SDIL by 2.13g per 100ml (95% CI: 2.08, 2.18).”

Reviewer 3

This study examines the impact of the U.K. Soft Drinks Industry Levy (SDIL), a tiered beverage tax based on sugar content, on tax pass-through, sugar content of beverages with respect to the levy thresholds, product size of available soft drinks, and availability of soft drinks. The authors draw on two different data sources that provide information on food and beverage products available for purchase on supermarket websites each week. The study uses an interrupted time series approach. The authors do not have a control site; however, they do make comparisons to a counterfactual based on the extrapolation of pre-announcement trends. It is critically important to understand the extent to which beverage taxes are passed on to consumers in the form of higher prices, the extent to which tiered taxes can incentivize reformulation, and the extent that the supply of beverages in the market place changes post-tax; hence, this study addresses important questions. However, the contribution of this study is severely limited by a number of queries and concerns related to the data and the lack of rigor in the specification an estimation of the empirical models which leads to significant distrust in the validity and interpretation of the results.

1.	Page 4, line 92. The paper states that soft drink taxes introduced elsewhere are normally valorem taxes. This is not true. Many are specific excise taxes levied on a per unit basis. See the following UNC website which provides an overview of the different types of beverage taxes worldwide: https://www.dropbox.com/s/bqbj501wgocor24/UNCGFRP_SSB_tax_maps.pdf?dl=0

*Response:* We agree with the reviewer – this was a mistake on our behalf. We have amended the manuscript to refer to previous soft drink taxes as excise taxes.

2.	Page 5, lines 105-106. The authors state that no soft drink tax that incentivizes industry to reduce sugar content - i.e., tired taxes - have been evaluated. There are at least two published evaluations of Chile's effective tiered tax.

*Response:* We agree that this was very clumsily worded on our part. We were trying to emphasise that the SDIL made reformulation of sugar drinks the prime target (rather than changes in consumer behaviour to reduce purchasing of taxed products). We have changed this statement to “Previous evaluations of soft drink taxes have focussed on their impact on price and consumer purchasing behaviour [22,23,24,25], but have not evaluated their impact on sugar content in drinks.”

3.	Page 6, line 131. The authors often refer to the "number" of soft drinks of available. The number may be confused with extent or depth of stock available which is not what is being assessed. Please be careful on wording.

*Response:* We have tried to clarify this statement as follows: “The number of soft drinks available for purchase from UK supermarkets. Here we refer to the different options available to the consumer, rather than the number of sales or the number of items available on supermarket shelves.”

4.	Page 7, lines 154-156. The authors note that the controls were chosen because it was assumed that they would not be affected. But they are substitutes and hence will have cross-price effects.

*Response:* We do not measure the impact of the SDIL on consumer purchasing behaviour, and hence there cannot be any cross-price *elasticity* effects. The reviewer may be referring to the fact that manufacturers may adjust price of substitute products in reaction to the SDIL, which is a limitation of our choice of control. We have added this to the limitations section of the discussion: “The control series may not be isolated from effects of the SDIL (e.g. manufacturers may choose to adapt prices of control drinks in response to the SDIL since they are a potential substitute for intervention drinks).”

5.	The foodDB data set consists of weekly data from November 2017 to February 2019. This does not even provide one full year of pre-tax data. There will be issues of seasonality. This is not at all addressed in the methods section. The analyses for price, product size, and availability of products was limited to this data set.

*Response:* Previous evaluations of soft drink taxes have demonstrated that it is important to account for seasonality in time series data for soft drink *purchases,* but our analyses are not based on purchasing behaviour. For each of our analyses, we observed the raw data to assess the trends within the dataset (we have added a section to the methods section describing this process in response to reviewer 1). We did not observe any seasonality in the sugar content and product size of soft drinks or in the availability of products. However, for price we observed a notable dip in soft drink prices in the month of December, due to retailers reducing prices over the Christmas period. Therefore we introduced a dummy variable for December to account for this seasonality in the price analyses but otherwise did not adjust for seasonality.

6.	There were inadequate descriptives given on the data. How many unique products? What about classifications by drink type - soda, energy drinks etc.

*Response:* We have added a section to the methods describing why it is not possible to identify unique products in this dataset (and see response to reviewer 2). A full description of the dataset in table 1 is not straightforward as there are five distinct analysis datasets that are used in this analysis, which are all related but not identical (see new data flowchart). Therefore, we have added a full descriptive table to appendix S1, based on the comparison dataset which contains overlapping data from both BrandView and foodDB.

7.	The methods do not provide any discussion on how the samples were constructed and whether they were balanced over time. Related to this is a significant concern about the fact that as beverages are reformulated over time they will actually jump across tax tiers. Thus, there is a concern about the composition of drinks changing within tiers over time. Much of what you are reporting say for tax pass-through by levy category may end up being an artifact of changing composition rather than within product effects. How do results change when the samples are balanced?

*Response:* Our original manuscript did not clearly describe the structure of the dataset, how data were collected, how the different analysis datasets were constructed and exclusions to the dataset. We hope that we have significantly improved the manuscript through clearer descriptions in a restructured methods section and through the additional data flowchart. With regard to this comment, we believe that our description in the original manuscript has led to a misunderstanding of our analyses. Our dataset is a repeat cross-sectional dataset, with a near comprehensive collection of data on all soft drinks available in UK online supermarkets over multiple time points between September 2015 and February 2019. Crucially, we have no unique identifying variable that can be used to link the soft drinks through time, therefore we are unable to analyse this dataset as panel data (which is implied by the reviewer’s references to balanced samples). We have clarified this in the methods section and to make this absolutely clear, we have added the following statement to the abstract: “The dataset was structured as a repeat cross-sectional study”. However, the reviewer’s concern regarding artefacts in the analysis of price pass-through is not possible as for both the price and product size analyses we restricted the datasets to those soft drinks that we could match with observations after the SDIL had been implemented (matching by name, which resulted in considerable loss of data). We then categorised each drink in the levy category that it occupied *after* the implementation of the SDIL (only for the price and product size analyses). This means that the drinks could not change levy category during the time period for these analyses. This process is described in detail in the restructured methods section.

8.	Related to the point above -- how do results change when product fixed effects are used? What product characteristics are controlled for in the regressions (it appears none from the appendices)? How is seasonality addressed? Do you include controls which supermarket the observation comes from? This is all critical to the model specification and not at all dealt with in the paper.

*Response:* These points are also related to the misunderstanding regarding the structure of the dataset, for which we apologise. In response to reviewer 1 we have also added sections to both the methods and the discussion where we explicitly state that our analyses are data-driven and aimed at reproducing and describing trends that we observe in the dataset, as opposed to isolating the effect of the SDIL on an average drink, after adjustment for product and supermarket characteristics. To reinforce this point we have added the following statement to the discussion section: “our aim was to reproduce trends observed in the UK over the time period studied using a near-comprehensive dataset of drinks available for purchase, but we did not aim to isolate the independent effect of the SDIL on an ‘average’ drink adjusted for product and supermarket characteristics. As a result, it is unlikely that the magnitude of our results will be generalizable to other jurisdictions considering introducing a similar levy.” Taking each point in turn: We cannot conduct analyses with observations nested in unique products as we have no unique product identifier; we do not control for any product characteristics in the analyses beyond conducting stratified analyses by branded status, as we are attempting to reproduce observed trends rather than isolate independent effects of the SDIL on an ‘average’ drink; our methods for addressing seasonality have been addressed in the response to a previous comment; we have not controlled for the supermarket from which the observations are taken, for the same reason as we have not controlled for product-level characteristics.

9.	Also of concern is the fact that interrupted time series analysis requires identification of the autocorrelation process of the time series. What autocorrelation structure did the authors use and how was this tested? There is no mention of this in the paper.

*Response:* Due to the lack of a unique identifier within the dataset, it is not possible to analyse these data as panel series (balanced or unbalanced). Therefore, we are analysing the data as a repeat cross-sectional analysis. Whilst it is possible to incorporate autocorrelation in repeat cross-sectional analyses (Lebo & Weber, 2015), we are unaware of a statistical package that could allow such analyses to be conducted on our dataset. We therefore accept as a limitation that our analyses do not account for autocorrelation. However, as the aim of these analyses is to describe observed patterns over time, as opposed to inferring relationships between product, or supermarket-level variables with sugar content, price or product size, we do not believe this is a serious limitation. For one of our analyses, we were able to incorporate autocorrelation - the ‘number of products’ analysis (where the data are collapsed onto time point before analysis). We have examined the residuals of these models and found little evidence of autocorrelation, and we have reconducted the analyses by incorporating a lag term in the exposure to account for autocorrelation and updated the manuscript accordingly (no major changes in results or interpretation). We have also added the following statement to the discussion: “Due to the lack of a unique product identifier in the dataset, it was not possible to analyse these data as a panel series, and hence we were unable to account for the autocorrelation structure in any of the analyses with the exception of the ‘number of products’ analysis.”

Lebo MJ, Weber C. An effective approach to repeated cross-sectional design. *American Journal of Political Science,* 2015;59(1):242-258.
